# Supplementary material for: A Likelihood Approach for Real-Time Calibration of Stochastic Compartmental Epidemic Models
Source: PLoS Comput Biol. 2017 Jan 17;13(1):e1005257. doi: 10.1371/journal.pcbi.1005257 (PMC5240920; doi:10.1371/journal.pcbi.1005257)
Supplement: S1 File — (TAR.GZ) [file pcbi.1005257.s014.tar.gz › HSPH_Online-SI-Revision/IPoi09/n1-mild/n1-mild_BENCH_table8.pdf]

1	R0	{1.27677, 7.89375}	{1.93839, 14.369}	{{1.84021, 12.8385}, {2.06384, 16.6365}}	{51.8197, 82.0295}	inf	4.	37.5185	{10., 74.}	837.963	0.858093	newly	2.	16.537	{4., 35.}	726.852	0.78812	Reff	0.856713	0.500469	{0.362147, 0.669109}	42.5354	ar	400.	781.111	{730., 855.}	95.2778
2	R0	{1.33231, 10.5259}	{2.20394, 15.6159}	{{2.07206, 14.2406}, {2.35866, 16.9062}}	{65.4227, 48.3568}	inf	6.	7.53968	{1., 16.}	81.746	0.415411	newly	4.	2.93651	{0., 6.}	66.2698	0.583646	Reff	0.714118	0.373892	{0.298642, 0.444969}	47.6429	ar	467.	833.905	{801., 869.}	78.5663
3	R0	{1.2273, 6.76944}	{1.67041, 7.56029}	{{1.58886, 7.28757}, {1.70777, 8.49961}}	{36.1046, 11.6826}	inf	9.	10.5897	{1., 31.}	100.285	0.529597	newly	6.	8.10256	{0., 25.}	112.821	0.593079	Reff	0.857883	0.559356	{0.443763, 0.671505}	35.1311	ar	314.	678.077	{624., 738.}	115.948
4	R0	{1.41215, 17.5742}	{1.56954, 11.3772}	{{1.53432, 10.5556}, {1.59621, 12.6816}}	{11.1458, 35.2621}	inf	25.	13.8108	{2., 42.}	73.2973	0.632347	newly	8.	7.97297	{1., 24.}	81.4189	0.472324	Reff	1.09159	0.602177	{0.495854, 0.750478}	44.835	ar	465.	641.189	{589., 691.}	37.8901
5	R0	{1.42222, 6.15886}	{2.27468, 14.9336}	{{2.18932, 14.058}, {2.41197, 16.0321}}	{59.9383, 142.474}	inf	11.	29.5385	{9., 56.}	177.762	0.37901	newly	5.	12.2923	{3., 23.}	163.077	0.376867	Reff	0.742399	0.361038	{0.27909, 0.431751}	51.3687	ar	490.	856.831	{823., 888.}	74.8634
6	R0	{1.24363, 9.95596}	{1.92576, 12.3934}	{{1.83169, 10.9611}, {2.03831, 13.7179}}	{54.8497, 24.523}	inf	11.	53.8036	{24., 96.}	390.097	0.637017	newly	12.	26.4107	{11., 46.}	125.446	0.314786	Reff	0.863079	0.53982	{0.422762, 0.707294}	37.4542	ar	411.	772.911	{727., 820.}	88.0561
7	R0	{1.36478, 5.96056}	{2.15929, 13.0857}	{{1.98732, 12.0157}, {2.28604, 14.464}}	{58.215, 119.539}	inf	8.	33.1379	{9., 68.}	318.534	0.512913	newly	5.	15.7069	{4., 32.}	223.793	0.420173	Reff	0.754723	0.405282	{0.308486, 0.522627}	47.4039	ar	484.	836.414	{803., 875.}	72.8128
8	R0	{1.36335, 17.6429}	{1.77289, 19.4236}	{{1.75588, 19.2972}, {1.79789, 19.6311}}	{30.0393, 10.0929}	inf	28.	65.449	{28., 116.}	136.516	0.335355	newly	12.	21.8776	{8., 39.}	95.5782	0.269835	Reff	1.02251	0.748146	{0.469249, 1.14659}	32.939	ar	484.	723.082	{672., 775.}	49.397
9	R0	{1.26575, 5.28707}	{1.98983, 19.5637}	{{1.98971, 19.0665}, {1.98971, 19.6444}}	{57.2055, 270.03}	inf	6.	63.4167	{24., 115.}	956.944	0.961342	newly	5.	21.2917	{7., 38.}	329.167	0.572945	Reff	0.669582	0.535829	{0.387001, 0.666553}	21.7625	ar	472.	794.271	{740., 835.}	68.2777
10	R0	{1.42942, 9.49905}	{2.27408, 19.0428}	{{2.23915, 18.4504}, {2.30371, 19.5287}}	{59.0911, 100.471}	inf	1.	22.5556	{7., 41.}	2155.56	1.19424	newly	1.	7.44444	{1., 13.}	650.794	0.724111	Reff	0.760451	0.363279	{0.276445, 0.47326}	53.4295	ar	468.	856.413	{824., 884.}	82.9942
11	R0	{1.46827, 15.0983}	{1.75406, 19.2561}	{{1.73196, 18.9248}, {1.75837, 19.4054}}	{19.4642, 27.5381}	inf	22.	69.7917	{33., 117.}	217.235	0.462162	newly	11.	24.6667	{9., 45.}	131.061	0.328759	Reff	1.12469	0.817973	{0.561882, 1.19049}	32.1457	ar	495.	703.958	{654., 757.}	42.2138
12	R0	{1.25751, 13.279}	{1.74813, 19.2342}	{{1.73196, 18.9248}, {1.75837, 19.4054}}	{39.0156, 44.8465}	inf	16.	69.	{36., 101.}	331.25	0.604806	newly	9.	25.4038	{14., 43.}	182.265	0.411638	Reff	0.975828	0.777093	{0.539749, 1.15522}	28.702	ar	366.	709.635	{662., 762.}	93.8892
13	R0	{1.3425, 9.16361}	{2.06284, 13.8936}	{{1.96814, 13.0262}, {2.16755, 14.8261}}	{53.6567, 51.6166}	inf	2.	11.3621	{1., 31.}	487.069	0.662029	newly	0.1	5.08621	{0., 14.}	5017.24	1.34108	Reff	0.753143	0.413397	{0.335172, 0.508198}	45.1104	ar	442.	810.069	{754., 846.}	83.2735
14	R0	{1.18936, 6.20583}	{1.98963, 11.9978}	{{1.91302, 11.1772}, {2.11292, 12.9719}}	{67.2856, 93.3316}	inf	3.	22.1346	{6., 42.}	637.821	0.681172	newly	3.	11.5962	{2., 16.}	301.923	0.474992	Reff	0.735024	0.462966	{0.371629, 0.538564}	39.8544	ar	382.	791.788	{759., 825.}	107.274
15	R0	{1.27137, 9.78868}	{1.99685, 14.4638}	{{1.94617, 13.1357}, {2.08951, 15.9744}}	{57.063, 47.7601}	inf	16.	50.7368	{21., 101.}	222.149	0.445781	newly	13.	20.7544	{8., 39.}	82.5911	0.256709	Reff	0.752651	0.492745	{0.363934, 0.654869}	35.6489	ar	496.	801.474	{752., 843.}	61.5874
16	R0	{1.23102, 13.6452}	{1.77731, 13.9486}	{{1.73739, 13.0272}, {1.83921, 14.6061}}	{44.3774, 4.56175}	inf	26.	39.0408	{10., 83.}	86.8917	0.29838	newly	13.	17.7347	{3., 37.}	80.6907	0.322383	Reff	0.853097	0.609812	{0.464086, 0.786482}	29.6979	ar	445.	714.163	{654., 764.}	60.4861
17	R0	{1.27213, 13.7452}	{1.67577, 12.2642}	{{1.64295, 11.6019}, {1.75163, 13.7237}}	{31.7293, 11.1264}	inf	30.	32.1053	{8., 84.}	68.0702	0.319769	newly	16.	17.8421	{3., 43.}	74.8904	0.354382	Reff	0.957914	0.680666	{0.478689, 0.989056}	32.6156	ar	367.	662.842	{589., 747.}	80.6109
18	R0	{1.25318, 11.2151}	{1.85345, 19.1738}	{{1.8189, 18.8228}, {1.87294, 19.9256}}	{47.8998, 70.964}	inf	24.	60.7241	{27., 97.}	154.454	0.366551	newly	17.	21.	{9., 36.}	47.4645	0.180559	Reff	0.833365	0.65021	{0.453251, 0.903332}	31.0755	ar	416.	747.655	{696., 794.}	79.7248
19	R0	{1.46006, 8.11496}	{2.3099, 14.8028}	{{2.18932, 13.5121}, {2.46246, 16.0321}}	{58.2058, 82.4141}	inf	20.	35.0781	{13., 57.}	91.1719	0.257873	newly	12.	14.2188	{5., 26.}	58.5938	0.242325	Reff	0.762151	0.349836	{0.265946, 0.428911}	54.0989	ar	496.	866.094	{831., 902.}	74.6157
20	R0	{1.14273, 6.71261}	{1.97537, 15.7601}	{{1.87975, 14.1819}, {2.05052, 16.7932}}	{72.864, 134.783}	inf	15.	52.3654	{17., 102.}	253.462	0.46817	newly	11.	20.4808	{6., 38.}	108.566	0.294433	Reff	0.742775	0.526256	{0.393203, 0.682349}	34.1858	ar	362.	790.385	{755., 828.}	118.338
21	R0	{1.42432, 8.23905}	{1.85163, 13.1203}	{{1.81743, 12.8385}, {1.94617, 14.0718}}	{30.0011, 59.2452}	inf	6.	22.2105	{5., 49.}	282.456	0.492342	newly	5.	10.5965	{1., 21.}	146.316	0.435318	Reff	0.853168	0.524162	{0.39118, 0.620738}	41.3027	ar	405.	750.807	{686., 805.}	85.3844
22	R0	{1.29386, 17.863}	{1.70609, 14.7757}	{{1.64814, 13.1646}, {1.79724, 16.8903}}	{31.8603, 17.4136}	inf	35.	52.1633	{24., 94.}	63.965	0.20494	newly	16.	23.7347	{10., 40.}	64.4133	0.212614	Reff	0.98204	0.765254	{0.531333, 1.23687}	32.082	ar	488.	688.714	{619., 753.}	41.13
23	R0	{1.39274, 13.0167}	{1.99593, 19.0309}	{{1.93087, 18.765}, {2.036, 19.6444}}	{43.3094, 46.2036}	inf	19.	27.0702	{9., 55.}	81.6251	0.282548	newly	8.	9.29825	{2., 21.}	70.1754	0.343878	Reff	0.799433	0.466467	{0.35528, 0.581192}	41.6503	ar	475.	790.632	{760., 837.}	66.4488
24	R0	{1.32178, 15.8991}	{1.99595, 19.3432}	{{1.98971, 19.0665}, {1.99771, 19.6444}}	{51.0045, 21.6624}	inf	28.	79.0385	{36., 131.}	184.203	0.40518	newly	13.	25.7692	{11., 42.}	106.213	0.273803	Reff	0.810251	0.590094	{0.413526, 0.819761}	30.0203	ar	471.	795.885	{758., 835.}	68.9776
25	R0	{1.37568, 17.8427}	{1.66899, 15.024}	{{1.64552, 13.8621}, {1.70497, 16.3716}}	{21.3212, 15.7976}	inf	24.	32.6522	{5., 65.}	79.1667	0.330778	newly	8.	13.8043	{2., 27.}	107.88	0.400329	Reff	1.00837	0.64836	{0.491031, 0.790845}	35.9379	ar	398.	667.739	{602., 730.}	67.7737
26	R0	{1.20993, 6.4656}	{1.88858, 14.4145}	{{1.82711, 13.6186}, {1.97176, 15.6958}}	{56.09, 122.941}	inf	2.	31.5306	{8., 65.}	1478.57	1.08579	newly	1.	13.6939	{3., 31.}	1273.47	1.02627	Reff	0.803394	0.500398	{0.402098, 0.637235}	39.4902	ar	345.	769.531	{726., 805.}	123.052
27	R0	{1.20548, 13.8003}	{1.86219, 19.2072}	{{1.8189, 18.8228}, {1.87294, 19.9256}}	{54.4773, 39.1793}	inf	23.	60.7708	{27., 107.}	165.489	0.382796	newly	10.	20.6458	{9., 36.}	112.292	0.292651	Reff	0.817315	0.61632	{0.421412, 0.818475}	28.2868	ar	350.	752.438	{695., 805.}	114.982
28	R0	{1.3176, 14.6594}	{1.65865, 14.7865}	{{1.5767, 12.7642}, {1.70497, 16.3716}}	{25.8842, 7.97587}	inf	16.	55.5	{20., 98.}	253.783	0.510813	newly	8.	24.0526	{6., 42.}	211.842	0.472586	Reff	1.06989	0.843033	{0.581306, 1.21662}	27.8842	ar	362.	659.526	{586., 717.}	82.1896
29	R0	{1.28202, 6.23047}	{2.14869, 18.4743}	{{1.98261, 16.3049}, {2.26107, 19.9579}}	{67.6022, 196.515}	inf	4.	47.2586	{16., 108.}	1081.47	0.957563	newly	5.	16.3448	{5., 36.}	230.345	0.416106	Reff	0.782032	0.441629	{0.330116, 0.553594}	43.5281	ar	391.	826.397	{771., 869.}	111.355
30	R0	{1.28177, 8.83252}	{2.03093, 13.4723}	{{1.93231, 12.4507}, {2.12155, 14.6763}}	{58.4471, 52.5307}	inf	18.	26.2295	{9., 56.}	76.5027	0.251255	newly	11.	11.9344	{3., 27.}	59.4635	0.291616	Reff	0.76778	0.440983	{0.379126, 0.508297}	42.5661	ar	427.	804.459	{775., 834.}	88.3979
31	R0	{1.35886, 5.97708}	{2.43252, 16.7291}	{{2.315, 15.4658}, {2.53519, 17.5902}}	{79.012, 179.887}	inf	0.001	34.8429	{11., 63.}	3.48419×10^6	2.43507	newly	0.1	12.6	{4., 23.}	12500.	1.99578	Reff	0.69166	0.328979	{0.237097, 0.428275}	52.4363	ar	491.	881.343	{842., 911.}	79.4996
32	R0	{1.27601, 17.9804}	{1.79679, 18.8236}	{{1.70573, 16.6329}, {1.84468, 19.9256}}	{40.8129, 7.50176}	inf	36.	66.4286	{31., 106.}	92.3611	0.27122	newly	15.	22.1429	{11., 34.}	64.0476	0.22227	Reff	0.962112	0.77637	{0.503986, 1.12044}	27.5805	ar	445.	722.214	{661., 786.}	62.2953
33	R0	{1.3368, 11.3235}	{2.21933, 19.2585}	{{2.12588, 18.765}, {2.26161, 19.6625}}	{66.0178, 70.0758}	inf	13.	72.9118	{35., 128.}	460.86	0.698019	newly	7.	23.6618	{9., 44.}	239.286	0.468408	Reff	0.788712	0.493592	{0.339568, 0.643662}	41.3217	ar	417.	840.588	{807., 875.}	101.58
34	R0	{1.21021, 11.3635}	{1.59144, 11.4663}	{{1.57414, 10.8293}, {1.64295, 12.6816}}	{31.5015, 6.03704}	inf	18.	15.9459	{2., 37.}	72.0721	0.442835	newly	15.	8.81081	{1., 21.}	66.8468	0.592488	Reff	0.905237	0.64418	{0.516348, 0.815405}	29.9474	ar	327.	628.595	{571., 689.}	92.2308
35	R0	{1.53382, 10.8866}	{2.26123, 19.3482}	{{2.2194, 19.1478}, {2.27287, 19.5128}}	{47.4248, 77.7246}	inf	21.	81.1563	{39., 129.}	287.649	0.535418	newly	15.	26.2188	{11., 47.}	87.9167	0.247694	Reff	0.852804	0.483281	{0.341503, 0.645845}	45.3083	ar	476.	850.313	{822., 881.}	78.6371
36	R0	{1.20423, 6.83844}	{2.15087, 12.7317}	{{2.04871, 11.6322}, {2.22518, 13.5548}}	{78.6093, 86.1787}	inf	0.001	22.913	{5., 52.}	2.2912×10^6	2.20016	newly	0.1	10.3043	{1., 26.}	10213.	1.77568	Reff	0.70327	0.389016	{0.303385, 0.473901}	44.6848	ar	416.	832.478	{799., 864.}	100.115
37	R0	{1.43629, 10.1548}	{1.88513, 15.8604}	{{1.85253, 15.8132}, {1.8951, 16.0085}}	{31.2498, 56.1867}	inf	12.	31.5185	{7., 70.}	182.407	0.39283	newly	8.	12.963	{3., 28.}	102.778	0.351073	Reff	0.963751	0.529392	{0.40972, 0.629173}	46.1186	ar	367.	759.648	{697., 803.}	106.989
38	R0	{1.37504, 8.93428}	{2.35741, 15.1087}	{{2.2143, 14.0824}, {2.47422, 16.1449}}	{71.4427, 69.1098}	inf	7.	28.4531	{10., 59.}	309.152	0.504532	newly	5.	11.7344	{2., 27.}	157.813	0.384932	Reff	0.717771	0.3384	{0.264742, 0.413195}	52.854	ar	483.	869.672	{836., 898.}	80.0563
39	R0	{1.51637, 9.36742}	{1.94495, 12.1463}	{{1.83592, 11.1369}, {2.04669, 13.0262}}	{28.2635, 29.6653}	inf	9.	11.8148	{3., 24.}	78.6008	0.335762	newly	4.	6.	{1., 12.}	94.4444	0.392703	Reff	0.884044	0.441474	{0.372692, 0.506714}	50.0619	ar	441.	781.778	{739., 815.}	77.2739
40	R0	{1.23658, 9.82869}	{2.07254, 12.0033}	{{1.98131, 11.1772}, {2.1245, 12.644}}	{67.6023, 22.125}	inf	1.	20.5806	{5., 45.}	1958.06	1.12721	newly	1.	10.371	{2., 23.}	946.774	0.849694	Reff	0.724636	0.408228	{0.322924, 0.512178}	44.2183	ar	414.	817.435	{766., 854.}	97.4482
41	R0	{1.24247, 5.4242}	{1.88135, 9.36412}	{{1.85533, 9.18466}, {1.93539, 9.64595}}	{51.4198, 72.636}	inf	5.	14.2807	{3., 28.}	203.86	0.420781	newly	5.	8.89474	{1., 18.}	121.404	0.389039	Reff	0.821273	0.514491	{0.406287, 0.625246}	40.2374	ar	345.	750.404	{702., 797.}	117.508
42	R0	{1.28208, 4.40223}	{1.92149, 9.59527}	{{1.85533, 9.18466}, {1.95791, 9.82151}}	{49.8732, 117.964}	inf	0.001	8.29091	{0., 19.}	829013.	1.61736	newly	0.1	4.78182	{0., 12.}	4714.55	1.33091	Reff	0.847455	0.448242	{0.372003, 0.518846}	47.1073	ar	339.	774.109	{738., 810.}	128.351
43	R0	{1.56773, 6.50474}	{2.35829, 9.96756}	{{2.22376, 9.35432}, {2.47628, 10.2848}}	{50.4272, 53.2353}	inf	3.	8.22727	{1., 18.}	210.606	0.43818	newly	2.	4.48485	{0., 10.}	193.939	0.583771	Reff	0.790136	0.320477	{0.24575, 0.389786}	59.4402	ar	498.	867.773	{834., 895.}	74.2516
44	R0	{1.10489, 6.01941}	{1.92424, 15.0712}	{{1.87975, 14.1819}, {2.00049, 16.2766}}	{74.1565, 150.376}	inf	1.	23.9574	{6., 54.}	2295.74	1.24148	newly	0.1	10.3191	{2., 26.}	10227.7	1.82372	Reff	0.724808	0.456293	{0.384192, 0.528129}	37.0463	ar	346.	782.085	{754., 815.}	126.036
45	R0	{1.25888, 14.1926}	{2.09084, 19.2}	{{2.036, 18.765}, {2.12588, 19.6444}}	{66.0872, 35.2815}	inf	17.	32.2542	{13., 53.}	99.1027	0.261543	newly	7.	10.678	{4., 20.}	75.3027	0.235607	Reff	0.682313	0.454579	{0.333904, 0.586368}	40.3195	ar	471.	819.847	{768., 860.}	74.0653
46	R0	{1.45292, 16.616}	{1.79717, 12.6053}	{{1.72065, 11.7288}, {1.84021, 13.6186}}	{23.6939, 24.1378}	inf	28.	35.4528	{7., 69.}	71.3612	0.301859	newly	9.	17.5094	{4., 37.}	120.545	0.334195	Reff	1.03012	0.589962	{0.425089, 0.760078}	43.8298	ar	494.	725.566	{664., 777.}	46.8757
47	R0	{1.22528, 16.6033}	{1.57225, 14.5556}	{{1.514, 12.7726}, {1.63728, 17.4454}}	{28.3175, 14.7444}	inf	30.	49.5417	{18., 79.}	82.3611	0.259296	newly	10.	22.9375	{8., 37.}	135.208	0.336836	Reff	1.00963	0.917313	{0.627943, 1.3595}	24.229	ar	368.	608.188	{549., 673.}	65.2683
48	R0	{1.1669, 6.92715}	{1.94083, 17.7895}	{{1.85253, 15.8132}, {1.99771, 19.2013}}	{66.3232, 156.809}	inf	6.	54.6415	{12., 119.}	811.321	0.831371	newly	3.	18.1509	{4., 40.}	511.321	0.66656	Reff	0.735147	0.538822	{0.425474, 0.68921}	27.5528	ar	416.	780.208	{735., 833.}	87.5499
49	R0	{1.32749, 16.0887}	{1.61567, 14.1058}	{{1.54363, 12.7642}, {1.68078, 15.8627}}	{21.7084, 12.8039}	inf	18.	38.5102	{9., 75.}	138.435	0.367319	newly	3.	17.6327	{3., 41.}	497.279	0.690443	Reff	1.06863	0.704569	{0.522723, 1.04504}	35.3185	ar	353.	655.571	{585., 712.}	85.7143
50	R0	{1.29636, 15.2609}	{2.09497, 19.35}	{{2.036, 18.765}, {2.12588, 19.5406}}	{61.6037, 26.7949}	inf	33.	53.7797	{21., 110.}	81.4587	0.241243	newly	13.	18.1695	{6., 35.}	72.6206	0.250714	Reff	0.735036	0.486708	{0.340476, 0.657187}	37.9018	ar	470.	817.78	{770., 858.}	73.9957
